# Replacement of dietary corn with corn bran plus condensed distillers solubles effects on feedlot growth performance and carcass trait responses of beef steers

**DOI:** 10.1093/tas/txac128

**Published:** 2022-09-09

**Authors:** Forest L Francis, Mallorie F Wilken, Zachary K Smith

**Affiliations:** Department of Animal Science, South Dakota State University, Brookings, SD 57007, USA; ICM, Inc., Colwich, KS 67030, USA; Department of Animal Science, South Dakota State University, Brookings, SD 57007, USA

**Keywords:** biorefinery, bran, coproducts, energetics, feedlot, fiber

## Abstract

Dry-corn milling biorefineries have the opportunity to install technology to fractionate corn prior to fermentation, which creates a product stream of fibrous bran that can be fed to cattle. The objective of this study was to determine the effects of replacing dietary corn with corn bran and condensed distillers solubles (CBCDS) or wet-corn gluten feed (WCGF) on growth performance, efficiency of dietary net energy (NE) utilization, and carcass characteristics in finishing steers. British × Continental steers (*n* = 240; initial body weight [BW] = 401 ± 43.2 kg) were assigned to the following dietary treatments in a randomized complete block design (RCBD): 1) a control finishing diet with no corn milling coproducts; 2) a finishing diet that contained CBCDS at 20% replacement of dietary corn; and 3) a finishing diet that contained WCGF at 20% replacement of dietary corn. Dietary corn (50:50 of dry-rolled corn and high-moisture corn) was included at 81.5% for control diet-fed steers and 61.5% for steers-fed CBCDS and WCGF. Steers were fed for 145.5 d until visually appraised to have 1.27 cm of rib fat (RF) and were harvested at a commercial abattoir where carcass data were collected. Data were analyzed as an RCBD with pen as the experimental unit, treatment as a fixed effect and block as a random effect. There were no significant differences (*P* ≥ 0.28) between treatments for final BW, average daily gain, dry matter intake, feed conversion efficiency, observed dietary NE for maintenance (NE_m_), and NE for gain (NE_g_), or observed-to-expected NE_m_ and NE_g_. Additionally, no differences (*P* ≥ 0.16) were noted between treatments for hot carcass weight, ribeye area, RF, marbling score, kidney–pelvic–heart fat, estimated empty body fat (EBF), BW at 28% EBF (AFBW), and distribution of USDA Quality and Yield grades. Control steers tended (*P* = 0.10) to have the highest calculated yield grade followed by WCGF and CBCDS. Furthermore, WCGF steers tended (*P* = 0.08) to have the highest calculated retail yield followed by CBCDS and control steers. Replacement NE_m_ and NE_g_ values of corn coproducts were determined to be 2.14 and 1.42 for CBCDS and 2.09 and 1.37 for WCGF, respectively. Thus, CBCDS can be included in finishing steer diets at 20% replacement of corn without detriment to growth performance or carcass characteristics.

## INTRODUCTION

In 2020, the United States produced 36.9 million metric tons of dry, modified, and wet distillers grains in addition to approximately 955,000 metric tons of condensed distillers solubles (CDS) as byproducts of ethanol production (RFA, 2021). Ethanol coproducts are an excellent source of cost-effective energy, protein, fat, fiber, and minerals which make them attractive to producers for use in livestock diets. As ethanol production increases and milling technologies advance, coproducts of the industry are becoming more intentionally designed to meet species-specific nutrient requirements and create additional revenue streams for the ethanol producers. New technology in the ethanol industry allows dry-corn milling biorefineries to fractionate corn fiber from fermentable corn constituent’s prefermentation resulting in greater ethanol yields for the facility (Sekhon et al., 2015). Due to the earlier fractionation of corn fiber in this process, the subsequently produced dried distillers grains (DDG) are more concentrated and have a higher crude protein content that fits specific parameters for use as a feed in nursery, growing, and finishing diets of pigs as a potential cost-effective replacement for soybean meal (Cemin et al., 2021; Rao et al., 2021). High-protein DDG have also been evaluated as a suitable feed in beef cattle feedlot diets (Garland et al., 2019). Additionally, these dry milling procedures fractionate corn fiber earlier in the biorefining process and it can be combined with CDS to create a new feed byproduct, corn bran plus condensed distillers solubles (CBCDS). Similarly, manufactured and quality products to CBCDS have been proven to be suitable feedstuffs in beef feedlot rations (Buckner et al., 2011; Garland et al., 2019). With varying nutrient composition and energy values of corn byproducts produced between biorefineries across the nation, it is common to test the novel feedstuffs as a dietary replacement for corn to determine their nutritional value as a diet ingredient. Thus, the objective of this experiment was to evaluate the influence that replacing dietary corn with a novel CBCDS has on finishing phase growth performance, efficiency of dietary net energy (NE) utilization, and carcass trait responses in beef steers.

## MATERIALS AND METHODS

The animal care and handling protocols used in this study were approved by the South Dakota State University Institutional Animal Care and Use Committee #1912-066E. This study was conducted at the South Dakota State University Ruminant Nutrition Center (RNC) in Brookings, South Dakota between 27 January 2020 and 22 June 2020.

### Dietary Treatments

This study used 30 pens of 8 steers per pen assigned to one of three dietary treatments in a randomized complete block design (RCBD). Dietary treatments included:

1. A finishing diet that contained no corn coproducts (CON);
2. A finishing diet that contained a dry-corn milling biorefinery product that replaced corn in the diet: corn bran plus CBCDS;
3. A finishing diet that contained a wet-corn milling coproduct that replaced corn in the diet: wet-corn gluten feed (WCGF).

Control diet-fed cattle received dietary corn (50:50 of dry-rolled corn [DRC] and high-moisture corn [HMC]) included in the diet at 81.5% (dry matter [DM] basis). Test ingredients evaluated in treatments 2 and 3 were included in the diet at 20% DM basis) as a replacement for dietary corn (50:50 DRC and HMC). Nutrient composition (DM basis) of the CBCDS and WCGF is in Table 1. The CBCDS was manufactured in four runs and delivered to the RNC and stored in a polypropylene agriculture bag. The WCGF was delivered on three occasions during the experiment and was stored under a commodity shed and covered with a plastic tarp.

**Table 1.** Nutrient composition (DM basis, except for DM) of the dry-corn milling biorefinery product (CBCDS) and wet-corn milling coproduct (WCGF) fed^a^

	Ingredient			
Item^b^	CBCDS	SD	WCGF	SD
DM, %	47.84	0.778	43.67	1.777
CP, %	27.69	1.054	20.48	1.127
NDF, %	37.07	1.745	43.20	2.081
ADF, %	12.06	1.823	12.34	1.045
EE, %	6.01	1.216	3.42	0.881
Ash, %	7.82	0.308	4.82	0.244
OM, %	92.18	0.308	95.18	0.244

Corn bran with condensed distillers solubles (CBCDS) and wet-corn gluten feed (WCGF).

Number of samples: DM (*n* = 21); CP (*n* = 16); NDF (*n* = 12); ADF (*n* = 12); EE (*n* = 21); Ash (*n* = 21); OM (*n* = 21).

### Study Initiation and Dietary Management

Two hundred and forty Continental × British crossbred steers (initial unshrunk body weight [BW] = 401 ± 43.2 kg) were used in an RCBD to evaluate the influence of dietary replacement of corn with CBCDS on finishing phase growth performance, efficiency of dietary NE utilization, comparative NE value, and carcass trait responses. All steers used in the experiment had previously been enrolled in separate receiving and backgrounding phase experiments conducted at the RNC. Steers had been vaccinated for viral respiratory pathogens (Bovi-Shield Gold 5, Zoetis, Parsippany, NJ), clostridial species (Ultrabac 7/Somubac, Zoetis), poured with moxidectin (Cydectin, Bayer Healthcare LLC, Shawnee Mission, KS) for the control of internal and external parasites, and administered a Synovex S (200 mg progesterone + 20 mg estradiol benzoate; Zoetis) approximately 90 d prior to initiation of the current study. All steers were transitioned to a common high-concentrate diet during a 21-d period (four steps) prior to the initiation of test diets. The common finishing diet that the steers were transitioned to prior to the initiation of the test diets was based upon (DM basis): dry-rolled corn (34%), high-moisture corn (34%), DDG plus solubles (20%), grass hay (7%), and a liquid supplement (5%). On January 27, 2020, all steers were individually weighed to collect a BW for allotment purposes and poured with cyfluthrin (Cylence, Bayer Healthcare LLC) for control of external parasites. The following day, January 28, 2020, the first five pen replicates for each treatment were individually weighed to collect an initial BW, and test diets were initiated. On January 29, 2020, the remaining five pen replicates for each treatment were individually weighed and test diets were initiated. Steers were implanted on day 14 with a Synovex PLUS (200 mg trenbolone acetate + 28 mg estradiol benzoate; Zoetis) and vaccinated for *Clostridium perfringens* (*C. perfringens* Type A toxoid, Elanco, Greenfield, IN).

Steers were housed in 58.06-m^2^ concrete surface pens with 7.62 m of linear bunk space and provided ad libitum access to feed; bunks were managed to be slick at 0700 h most mornings. Diets were fortified to provide vitamins and minerals to meet or exceed nutrient requirements (NASEM, 2016) and provided monensin sodium at 33.08 mg/kg of diet DM. Fresh feed was manufactured twice daily in a stationary horizontal mixer (2.35 m^3^; Roto-Mix, Dodge City, KS; scale readability ±0.45 kg) and offered to steers in a 50:50 split at 0800 and 1400 h. Individual ingredient samples were collected weekly and DM was calculated following drying in a 60 °C forced air oven until no weight change to calculate dry matter intake (DMI). Proximate analysis of ingredients was conducted weekly; DM (method no. 935.29 [AOAC, 2012]), N (method no. 968.06 [AOAC, 2016]; Rapid Max N Exceed, Elementar, Mt. Laurel, NJ), and ash (method no. 942.05 [AOAC, 2012]). Ether extract (EE) content analysis was conducted for CBCDS and WCGF utilizing an Ankom Fat Extractor (XT10; Ankom Technology, Macedon, NY); tabular values were used for the remainder of the ingredients (NASEM, 2016). Acid detergent fiber (ADF) and neutral detergent fiber (NDF) percentages were estimated to be 3% and 9%, respectively, for both DRC and HMC; fiber content analysis for all other ingredients was conducted as described by Goering and VanSoest (1970). Actual diet formulation was based on weekly DM analyses and corresponding feed batching records. Diets presented in Table 2 are actual nutrient concentrations, and tabular energy values (NASEM, 2016). Due to the depletion of HMC inventory on study day 101, diets were reformulated to have DRC replace HMC for the remainder of the experiment.

**Table 2.** Actual DM formulation and nutrient composition of diets fed calculated from weekly ingredient assays and batching records.^a^^,^^b^

	1 to 101			101 to end		
Item	CON	CBCDS	WCGF	CON	CBCDS	WCGF
Dry-rolled corn, %	40.79	30.52	30.70	81.69	61.55	61.15
High-moisture corn, %	40.68	30.59	30.76	-	-	-
Corn bran plus condensed distillers solubles, %	-	20.49	-	-	20.11	-
Wet-corn gluten feed, %	-	-	20.05	-	-	20.69
Meal supplement, %^c^	7.00	7.02	6.99	6.86	6.92	6.83
Grass hay, %	6.52	6.41	6.50	6.56	6.54	6.49
Liquid supplement, %^d^	5.01	4.97	5.00	4.89	4.88	4.84
Nutrient composition						
DM, %	78.83	69.66	68.10	87.33	74.73	72.57
CP, %	12.31	13.36	13.44	11.89	12.72	12.85
NDF, %	12.84	20.14	19.59	12.86	19.92	20.24
ADF, %	5.73	8.66	7.47	5.78	9.08	7.84
EE, %	3.76	4.22	3.75	3.77	4.29	3.80
Ash, %	4.82	6.31	5.88	4.76	6.09	5.70
NE_m_, Mcal/kg	2.11	2.10	2.09	2.07	2.07	2.07
NE_g_, Mcal/kg	1.41	1.41	1.40	1.39	1.39	1.38

All values except for DM on a DM basis.

Control diet containing no coproducts (CON); corn bran with condensed distillers solubles (CBCDS); wet-corn gluten feed (WCGF).

Meal supplement contained (DM basis): 87.50% soybean meal (54% CP), 2.85% trace mineralized salt, 2.85% urea, and 8.60% ground corn (CON); 42.85% soy bean hulls, 8.57% calcium carbonate, and 48.58% ground corn (CBCDS); 57.14% soybean meal (54% CP), 1.57% trace mineralized salt, 8.57% calcium carbonate, and 32.72% ground corn (WCGF).

Liquid supplement contained (DM basis): 44.18% CP, 38.97% non-protein nitrogen, 1.06 Mcal/kg of NE_m_, 0.73 Mcal/kg of NE_g_, 0.80% ether extract, 13.57% total sugars, 50.77% ash, 11.06% calcium, 0.32% P, 7.10% K, 0.22% Mg, 2.50% NaCl, 1.80% Na, 6.42% Cl, 0.38% S, 3.41 ppm Co, 202.94 ppm Cu, 12.18 ppm I, 15.3 mg/kg EDDI, 97.22 ppm Fe, 309.49 ppm Mn, 2.94 ppm Se, 674.78 ppm Zn, 44,741 IU/kg Vitamin A, 446.5 IU/kg Vitamin E, and 648.56 mg/kg monensin sodium.

### Health Management

All steers that were pulled from their home pen for health evaluation were then monitored in individual hospital pens prior to being returned to their home pens. When a steer was moved to a hospital pen, the appropriate amount of feed from the home pen was removed and transferred to the hospital pen. In instances where the steer in the hospital pen was returned to the home pen, its feed remained credited to the home pen. If the steer did not return to its home pen, all feed delivered to the hospital pen were deducted from the feed intake record for that pen back to the date the steer was hospitalized. One steer died in the CON treatment from issues related to the bovine respiratory disease complex. Two steers were removed from the WCGF treatment; one due to irresolvable bloat and one due to issues related to bovine respiratory disease complex. Additionally, one steer from the CON treatment was removed from the study due to musculoskeletal issues. The dead steer and three removals were determined to be health anomalies not related to dietary treatment.

### Study Termination, Harvest, and Carcass Data Collection

Cattle were weighed off test when they were visually appraised to have 1.27 cm of rib fat (RF). On the day of study termination, cattle were shipped 238 km to a commercial beef processor, and harvested the following morning. Steers were commingled at the time of study termination and remained as such until 0700 h in the morning of harvest. Individual steer identity was tracked through the harvest facility using electronic identification tags. Hot carcass weight (HCW) was recorded during the harvest procedure. Video image data were obtained from the plant for ribeye area (REA), RF, kidney–pelvic–heart fat (KPH), and USDA marbling scores. Dressed yield was calculated as: (HCW/final BW shrunk 4%) × 100. Estimated EBF percentage and final BW at 28% estimated empty body fatness (AFBW) were calculated from observed carcass traits (Guiroy et al., 2002). Yield grade (YG) was calculated according to the USDA regression equation (USDA, 2017). Estimated proportion of closely trimmed boneless retail cuts from the carcass round, loin, rib, and chuck (Retail Yield; RY) was also calculated from carcass traits (Murphey et al., 1960).

### Cattle Growth Performance Calculations

Growth performance was calculated on a deads- and removals-excluded basis. Following study initiation, steers were individually weighed on days 14, 42, 77, 105, and 145, or 146 for the calculation of cumulative average daily gain (ADG) and feed conversion efficiency (gain:feed; [G:F]). Steer performance was calculated with a 4% shrink applied to initial BW to account for gastrointestinal tract fill. Dressing percentage was calculated as follows: (Hot carcass weight [HCW] ÷ Final shrunk BW) × 100. Cumulative carcass-adjusted growth performance was calculated from: HCW ÷ 0.6433 (average dressed yield of all steers in study).

Carcass-adjusted growth performance was used to calculate performance-based dietary NE to determine the efficiency of dietary NE utilization. The performance-based dietary NE was calculated from daily energy gain (EG; Mcal/d): EG = (ADG)^1.097^ × 0.0493W^0.75^; where W is the median feeding shrunk BW calculated as: ([initial shrunk BW + carcass-adjusted final BW] ÷ 2). Maintenance energy (EM) was calculated by the equation: EM = 0.077 × median feeding shrunk BW^0.75^. Dry matter intake is related to energy requirements and dietary NE_m_ (Mcal/kg) according to the following equation: DMI = EG ÷ (0.877NE_m_ – 0.41), and can be resolved for estimation of dietary NE for maintenance (NE_m_) by means of the quadratic formula $x=\frac{-b\pm \sqrt{b^{2}-4ac}}{2c}$, where *a* = −0.41EM, *b* = 0.877EM + 0.41DMI + EG, and *c* = –0.877DMI (Zinn et al., 2008). Dietary NE for gain (NE_g_) was derived from NE_m_ using the following equation: NE_g_= 0.877NE_m_ – 0.41 (Zinn et al., 2008).

The comparative NE_m_ and NE_g_ values for CBCDS and WCGF were estimated using the replacement technique using the weighted NE values of DRC and HMC fed over the experiment (1.52 Mcal NE_m_/kg and 2.24 Mcal NE_g_/kg). The following equation was used to calculate the comparative NE_m_ and NEg values for CBCDS and WCGF:


$$\begin{array}{l} Test\;ingredient\;NE_{m}=([Test\;diet\;NE_{m}\;-CON\;diet\;NE_{m}]\;\\ ÷\;Test\;ingredient\;inclusion)+2.24 \end{array}$$

$$\begin{array}{l} Test\;ingredient\;NE_{g}=([Test\;diet\;NE_{g}\;-CON\;diet\;NE_{g}]\;\\ ÷\;Test\;ingredient\;inclusion)+1.52 \end{array}$$

### Statistical Analysis

Growth performance, carcass traits, and efficiency of dietary NE utilization were analyzed as an RCBD using the GLIMMIX procedure of SAS 9.4 (SAS Inst. Inc., Cary, NC) with pen as the experimental unit. Distribution of USDA yield and quality grades were analyzed as binomial proportions in the GLIMMIX procedure of SAS 9.4. Both models included the fixed effects of block and dietary treatment. Least squares means were generated using the LSMEANS statement of SAS 9.4. For all analyses, an *α* of 0.05 determined significance and an *α* of 0.06 to 0.10 was considered a tendency.

## RESULTS

Cumulative carcass-adjusted growth performance and efficiency of dietary NE utilization are in Table 3. No differences (*P* ≥ 0.58) among treatments were detected for carcass-adjusted final BW, ADG, or feed conversion efficiency. Observed dietary NE_m_ and NE_g_ were not impacted (*P* ≥ 0.28) by treatment. Additionally, no difference (*P* ≥ 0.40) was detected for the ratio of observed-to-expected dietary NE_m_ or NE_g_. Carcass trait responses are located in Table 4. There was no influence (*P* ≥ 0.16) of dietary treatment on dressing percentage, HCW, REA, RF, marbling score, KPH, estimated EBF, and AFBW. A tendency to differ (*P* = 0.10) was observed for calculated YG, where steers-fed CBCDS and WCGF had 4.3% and 6.1% lower YG than CON, respectively. Additionally, CBCDS and WCGF cattle tended (*P* = 0.08) to have about a 1% increase in calculated RY compared to CON. No differences (*P* ≥ 0.29) were observed between dietary treatments for the distribution of USDA quality and yield grading distributions. The comparative NE_m_ and NE_g_ values of the CBCDS and the WCGF using the replacement technique are located in Table 1. Given the dietary replacement of corn for 20.37% CBCDS and 20.25% WCGF, replacement NE_m_ and NE_g_ values (Mcal/kg) were determined to be 2.14 and 1.42 for CBCDS and 2.09 and 1.37 for WCGF.

**Table 3.** Cumulative shrunk growth performance responses, efficiency of dietary NE utilization, and estimated ingredient NE value.

	Dietary treatment^a^				
Item	CON	CBCDS	WCGF	SEM	*P* - value
Pens, *n*	10	10	10		
Steers, *n*	78	80	78		
Days on feed	145.5	145.5	145.5		
Growth performance^b^					
Initial BW, kg	386	385	385	1.04	0.78
Final BW, kg	640	637	634	5.22	0.77
ADG, kg	1.74	1.73	1.70	0.073	0.72
DMI, kg	10.69	10.76	10.67	0.13	0.89
G:F (ADG/DMI)	0.163	0.161	0.160	0.0022	0.58
Observed NE, Mcal/kg					
Maintenance	2.11	2.09	2.08	0.021	0.53
Gain	1.44	1.42	1.41	0.018	0.53
Observed and expected NE^c^					
Maintenance	1.01	0.99	1.00	0.015	0.80
Gain	1.03	1.02	1.01	0.013	0.56
Estimated NE value of corn milling coproduct, Mcal/kg^d^					
Maintenance	-	2.14	2.09	-	-
Gain	-	1.42	1.37	-	-

Control diet containing no coproducts (CON); corn bran with condensed distillers solubles (CBCDS); wet-corn gluten feed (WCGF).

Final BW calculated from HCW/0.6433 (the average dressing percentage of the study).

Tabular NE (Mcal/kg) for CON was 2.09 and 1.40 for maintenance and gain, respectively; for CBCDS was 2.09 and 1.40 for maintenance and gain, respectively; for WCGF was 2.08 and 1.40 for maintenance and gain, respectively. The tabular NE_m_ and NE_g_ for the corn coproducts was assumed to be the same as dry-rolled corn at 2.20 Mcal/kg NE_m_ and 1.50 Mcal/kg NE_g_.

The estimated NE_m_ and NE_g_ values for corn coproducts were estimated using the replacement technique. Given that the weighted NE_m_ and NE_g_ value of DRC and HMC that was displaced over the feeding period was 2.24 Mcal/kg NE_m_ and 1.52 Mcal/kg NE_g_. The following equation was used to calculate the comparative NE values for corn co-product: co-product NE_m_ or NE_g_, Mcal/kg = [(Co-product diet NE_m_ or NE_g_ – CON NE_m_ or NE_g_)÷y] + NE_m_ or NE_g_ of the corn that was displaced by the corn co-product, where y represents the inclusion of corn co-product that replaced corn in the diet (0.2037 for CBCDS and 0.2025 for WCGF, respectively).

**Table 4.** Carcass trait responses

	Dietary treatment^a^				
Item	CON	CBCDS	WCGF	SEM	*P* - value
Pens, *n*	10	10	10		
Steers, *n*	78	80	78		
Carcass traits					
Dressing percentage^b^, %	64.14	64.71	64.13	0.333	0.39
Hot carcass weight, kg	411	410	408	3.4	0.77
Ribeye area, cm^b^	97.29	98.13	97.00	0.735	0.68
Rib fat, cm	1.32	1.24	1.22	0.038	0.19
Marbling score^c^	487	479	473	8.8	0.54
Kidney–pelvic–heart fat, %	1.82	1.80	1.78	0.017	0.27
Calculated yield grade^d^	2.78^f^	2.66^f,g^	2.61^g^	0.056	0.10
Retail yield, %	50.97^g^	51.24^f,g^	51.35^f^	0.117	0.08
Estimated EBF, %^e^	30.16	29.71	29.46	0.252	0.16
Body weight adjusted to 28% EBF (AFBW), kg^e^	609	614	615	4.5	0.61
USDA quality grade,%					
Select, %	15.00	16.25	18.75	4.583	0.84
Low choice, %	47.38	46.25	48.33	4.629	0.95
Average choice, %	25.54	31.25	26.25	5.397	0.72
High choice, %	12.08	5.00	6.67	3.200	0.29
Prime, %	0.00	1.25	0.00	0.722	0.39
USDA yield grade, %					
Yield grade 1, %	6.25	12.50	17.50	5.320	0.35
Yield grade 2, %	51.61	50.00	51.67	4.409	0.96
Yield grade 3, %	38.21	35.00	29.58	3.773	0.29
Yield grade 4, %	3.93	2.50	1.25	1.694	0.55

Control diet containing no coproducts (CON); corn bran with condensed distillers solubles (CBCDS); wet-corn gluten feed (WCGF).

Calculated as hot carcass weight/final BW shrunk 4%.

400= Small^00^ (USDA low choice).

Calculated according to the USDA regression equation (USDA, 2017).

Calculated according to Guiroy et al. (2002).

Means within a row without a common superscript differ (*P* ≤ 0.10).

## DISCUSSION

As fractionation technology advances for dry milling ethanol production, there will continue to be a stream of new coproducts with varying nutrient composition produced by biorefineries. Additionally, the flow of products from dry and wet milling plants will differ significantly in nutrient composition. It is then crucial to determine how different coproducts from both the dry and wet milling industries perform in cattle diets in differing stages of production and how they interact with other feedstuffs in a total mixed ration. Garland et al. (2019) studied calf-fed steers consuming either dried distillers grains plus solubles (DDGS), high-protein DDGS, wet DGS (WDGS), or CBCDS at 40% inclusion in the diet. The authors reported the CBCDS steers had increased final BW, ADG, G:F, and HCW compared to all other corn milling coproducts, indicating that fractionated bran coproducts can be effectively fed to finishing steers. However, the nutrient composition of the CBCDS in Garland et al. (2019) differed from the CBCDS in the current study by having decreased DM content and NDF, increased CP, and differing fat content. Ganesan et al. (2008) reported that by increasing the CDS levels in DDGs from 15% to 20%, there was a 68% increase in the fat content of the DDGS. Steers-fed increasing levels of corn bran and steep have been reported to have increased DMI, ADG, and improved G:F, in addition to increased YG and RF compared to corn-fed controls (Scott et al., 1997). However, Rodenhuis et al. (2017) reported that in steers-fed low-oil DDGS and moderate-oil DDGS with both rolled corn and barley that there was no difference in growth performance or carcass characteristics for the oil level in DDGS. In the current study, the exact inclusion of CDS in the CBCDS and steep in the WCGF was unknown; however, the EE value of the CBCDS was 62% higher than the WCGF and would be expected to have a higher inclusion of CDS in the final product. Still, results at similar levels of bran inclusion to Scott et al. (1997) were not observed in the current study. Sayer et al. (2013) reported no difference in ADG or G:F and only a tendency for DMI to increase in cattle-fed corn bran with differing levels of steep inclusion in the replacement of corn during the winter and spring months. These results corroborate the findings of the current study where during winter and spring months, steers-fed CBCDS and WCGF had similar ADG, DMI, and G:F compared to CON cattle. According to Pritchard et al. (2012), the energy value of dry-corn milling coproducts is largely driven by the fat content of the ingredient; for each percentage point of fat increase of the coproduct, the NE_g_ of the coproduct increases 0.05 Mcal/kg. Based on the regression estimates generated by Pritchard et al. (2012) and the fat content of the coproducts fed in the present experiment (Table 1), the NE_g_ values of CBCDS and WGCF would be 96.5% and 88.8% of the NE_g_ value of DRC, respectively. The increased NE_g_ for CBCDS compared to WCGF estimated from regressed estimates from Pritchard et al. (2012) is due to the increased fat content of the CBCDS. The feeding values of CBCDS and WCGF based upon their fat content are in close agreement with the replacement NE_g_ value generated from performance-based NE values in this study. However, the nutrient composition of the CBCDS and WCGF (Table 1) cannot fully explain the NE differences between the corn milling coproducts and dietary corn. Net energy prediction equations using feedstuff composition from Zinn and Plascencia (1993) estimate the NE_g_ value of CBCDS and WCGF to be 1.39 Mcal/kg, approximately 93% of the NE_g_ value of DRC. The NE_g_ values from Zinn and Plascencia (1993) are estimated to be lower for CBCDS and higher for WCGF than replacement and fat-derived NE_g_ values. Net energy values predicted by Zinn and Plascencia (1993) differ from other estimates of ingredient NE likely because the equation accounts for ash content of the feed when calculating nitrogen-free extract content of the feed. The CBCDS had 162% the ash content of the WCGF, which effectively inflated the NE values of WCGF beyond replacement fat-derived estimates.

## CONCLUSION

The inclusion of CBCDS in finishing cattle diets in this study had no negative effect on growth performance or carcass characteristics; hence, CBCDS can be included at 20% of the diet (DM basis) in finishing rations as a replacement for corn with no appreciable influence on growth performance. Additionally, the NE_m_ and NE_g_ values of CBCDS used in this study were determined to be 2.14 and 1.42 Mcal/kg, respectively. With an increasing number of corn milling coproducts with varying nutrient composition, it will continue to be necessary to investigate the feeding value of products as they become available for use in beef feedlot rations.

## List of Abbreviations

ADF: acid detergent fiber
ADG: average daily gain
AFBW: final body weigh at estimated 28% empty body fat
BW: body weight
CBCDS: corn bran plus condensed distillers solubles
CDS: condensed distillers solubles
CON: diet with no corn coproducts
DDG: dried distillers grains
DDGS: dried distillers grains with solubles
DM: dry matter
DMI: dry matter intake
DRC: dry-rolled corn
EBF: empty body fat
EE: ether extract
EG: daily energy gain
EM: maintenance energy
G:F: gain:feed
HCW: hot carcass weight
HMC: high-moisture corn
KPH: kidney, pelvic and heart fat
NDF: neutral detergent fiber
NE: net energy
NE_m_: net energy for maintenance
NE_g_: net energy for gain
REA: ribeye area
RF: rib fat
RY: retail yield
WCGF: wet-corn gluten feed
WDGS: wet distillers grains with solubles
YG: yield grade
